# How to get maximum structure information from anisotropic displacement parameters obtained by three-dimensional electron diffraction: an experimental study on metal–organic frameworks

**DOI:** 10.1107/S2052252522005632

**Published:** 2022-06-08

**Authors:** Laura Samperisi, Xiaodong Zou, Zhehao Huang

**Affiliations:** a Department of Materials and Environmental Chemistry, Stockholm University, Stockholm, Sweden 106 91, Sweden

**Keywords:** anisotropic displacement parameters, ADPs, three-dimensional electron diffraction, 3D ED, continuous rotation electron diffraction, metal-organic frameworks

## Abstract

Three-dimensional electron diffraction (3D ED) has been used for *ab initio* structure determination of various types of nanocrystals, such as metal–organic frameworks (MOFs), zeolites, metal oxides and organic crystals. These crystals are often obtained as polycrystalline powders, which are too small for single-crystal X-ray diffraction (SCXRD). While it is now possible to obtain accurate atomic positions of nanocrystals by adopting kinematical refinement against 3D ED data, most new structures are refined with isotropic displacement parameters (*U*_eq_), which limits the detection of possible structure disorders and atomic motions. Anisotropic displacement parameters (ADPs, *U^ij^*) obtained by anisotropic structure refinement, on the other hand, provide information about the average displacements of atoms from their mean positions in a crystal, which can provide insights with respect to displacive disorder and flexibility. Although ADPs have been obtained from some 3D ED studies of MOFs, they are seldom mentioned or discussed in detail. We report here a detailed study and interpretation of structure models refined anisotropically against 3D ED data. Three MOF samples with different structural complexity and symmetry, namely ZIF-EC1, MIL-140C and Ga(OH)(1,4-ndc) (1,4-ndcH_2_ is naphthalene-1,4-dicarboxylic acid), were chosen for the studies. We compare the ADPs refined against individual data sets and how they are affected by different data-merging strategies. Based on our results and analysis, we propose strategies for obtaining accurate structure models with interpretable ADPs based on kinematical refinement against 3D ED data. The ADPs of the obtained structure models provide clear and unambiguous information about linker motions in the MOFs.

## Abstract 2

Structure models of metal–organic frameworks (MOFs) obtained after refining anisotropic displacement parameters (ADPs) against three-dimensional electron diffraction (3D ED) data can reveal important structural features, such as linker motion. We report strategies to obtain the maximum structure information from ADPs refined against 3D ED data.

## 1. Introduction

Metal–organic frameworks (MOFs) are a class of crystalline materials in which organic linkers connect metal nodes to form three-dimensional (3D) frameworks (Yaghi *et al.*, 1995 ▸; Kitagawa *et al.*, 2004 ▸). Their structures can display large-scale rotational dynamics (Horike *et al.*, 2006 ▸; Schneemann *et al.*, 2014 ▸; Bennett *et al.*, 2017 ▸; Morris & Brammer, 2017 ▸; Bigdeli *et al.*, 2020 ▸). Gate-opening effects, breathing, swelling, and molecular libration and rotation occur in a considerable variety of MOFs, and such effects have shown clear influences in gas sorption and separation (Kitaura *et al.*, 2003 ▸; Mohideen *et al.*, 2011 ▸; Mason *et al.*, 2015 ▸; Lin *et al.*, 2017 ▸; Su *et al.*, 2017 ▸; Gu *et al.*, 2019 ▸), diffusion of molecules (Fairen-Jimenez *et al.*, 2011 ▸; Murdock *et al.*, 2014 ▸; Katsoulidis *et al.*, 2019 ▸), optical properties (Dong *et al.*, 2007 ▸; Shustova *et al.*, 2011 ▸; Serra-Crespo *et al.*, 2012 ▸; Du *et al.*, 2018 ▸) and mechanical properties (Lock *et al.*, 2010 ▸, 2013 ▸; Peterson *et al.*, 2010 ▸; Collings *et al.*, 2014 ▸; Balestra *et al.*, 2016 ▸; Pang *et al.*, 2016 ▸; Wang *et al.*, 2016 ▸).

In crystallographic refinement, anisotropic displacement parameters (ADPs) are used to describe the thermal vibrations of atoms, which are affected by tem­per­ature. Moreover, ADPs can also be used to model the effects of static and dynamic disorder (Hummel *et al.*, 1990 ▸; Bürgi, 2000 ▸; Bürgi & Capelli, 2003 ▸; Samperisi *et al.*, 2021 ▸). Refinement of a structure model with isotropic displacement uses four independent parameters to describe each atom, three for coordinates (*x*, *y* and *z*) and one to model atomic displacement, *U*_eq_ (Trueblood *et al.*, 1996 ▸). A structure model refined anisotropically requires nine parameters, as the ADPs are modelled by six parameters (*U* ^11^, *U* ^12^, *U* ^13^, *U* ^22^, *U* ^23^ and *U* ^33^) which describe an ellipsoid. The correct interpretation of the ADPs is essential for analysing molecular flexibility, as the motion of molecules or of domains is an anisotropic phenomenon (Schomaker & Trueblood, 1968 ▸; Dunitz *et al.*, 1988 ▸).

The use of three-dimensional electron diffraction (3D ED) methods has grown rapidly over recent decades and it has proven to be particularly beneficial for the structure determination of MOFs (Gemmi *et al.*, 2019 ▸; Huang *et al.*, 2021*a* ▸,*b* ▸,*c* ▸; Ge *et al.*, 2021*a* ▸,*b* ▸,*c* ▸). These methods allow single-crystal structure determination of all the crystalline compounds co-existing in a powder sample, which is powerful for mixtures of submicron and nanocrystalline MOFs. 3D ED methods have demonstrated that they can elucidate atomic positions of MOF frameworks with an accuracy which is approaching that of well-established single-crystal X-ray diffraction (SCXRD) (Huang *et al.*, 2021*b* ▸). Despite the occurrence of dynamical effects, it has also been demonstrated that by adopting a kinematical approximation during refinement, accurate atomic positions in MOFs and hydrogen bonds (Wang *et al.*, 2017 ▸; Cui *et al.*, 2020 ▸; Xu *et al.*, 2020 ▸; Zhu *et al.*, 2022 ▸) can be obtained and fine structural features, such as the locations of guest molecules (Wang *et al.*, 2018 ▸), linker disorder (Leubner *et al.*, 2020 ▸) and long-range molecular dynamics (Samperisi *et al.*, 2021 ▸) of nanosized MOFs, can be detected. However, ADPs in MOF structures have been less explored by 3D ED. Although these parameters can reveal interesting phenomena, such as disorder and motions, ADPs are rarely discussed in the studies of MOF structures obtained by 3D ED.

SCXRD shows that the ellipsoids described by the ADPs are not only affected by static and dynamic disorder, but can also be a result of various kinds of model errors, such as incomplete data, noise, inadequate resolution, unaccounted diffuse scattering, deficiencies in the model of the scattering density, inaccuracy of the independent atomic model, twinning, charge density, use of different refinement protocols, *etc*. (Bürgi, 2000 ▸; Bürgi & Capelli, 2003 ▸; Kofoed *et al.*, 2019 ▸). When these errors find their way into ADPs, the displacement parameters can turn to negative values of the tensors and the corresponding atoms are described as non-positively defined (NPD). In other cases, the errors can give rise to positively definite ellipsoids with equally unreasonable physical meaning, like extremely flat, inflated or elongated ellipsoids. The scarcity of information regarding ADPs obtained by 3D ED methods for MOF structures might be attributed to a tendency towards preferring other validation strategies, like Rietveld refinement against powder X-ray diffraction (PXRD) data. The dynamical effects caused by the strong interaction between electrons and matter also affect the measured ED intensities, causing them to deviate from the kinematical intensities. However, 3D ED studies in which dynamical effects were taken into account in the structure refinement show that the abovementioned problems sometimes still remain (Palatinus *et al.*, 2015 ▸).

It is true that without taking coarse effects into account, ADPs alone can only provide simple models; nonetheless, good starting ellipsoidal models can reveal interesting structural features that are worthy of further investigation. We have recently shown that the structure models obtained after kinematical refinement of ADPs against 3D ED data can probe motion and disorder in MOFs with results comparable to those obtained from SCXRD and molecular dynamics simulations (Samperisi *et al.*, 2021 ▸). It was also reported that the ADPs could differ when refined against X-ray and neutron diffraction data from the same compounds (Flensburg *et al.*, 1995 ▸; Şerb *et al.*, 2011 ▸). Therefore, it is important to understand the conditions under which 3D ED can be used to obtain structural information from ADPs. Here we take a step further and describe our best practices for obtaining structure models with physically sensible ADPs, with a focus on data processing and structure refinement. This can provide a fundamental understanding of the structural complexity of MOFs. We apply a 3D ED protocol, namely, continuous rotation electron diffraction (cRED), to three MOFs with different structural characteristics. For all the selected examples, we carefully interpreted the obtained structure models from individual and merged data sets, and analysed the ADPs. The comparison of the results allows us to propose our best practices for obtaining physically meaningful ADPs and also to evaluate the current limitations of 3D ED.

## 2. Investigated structures

**Figure 1:**
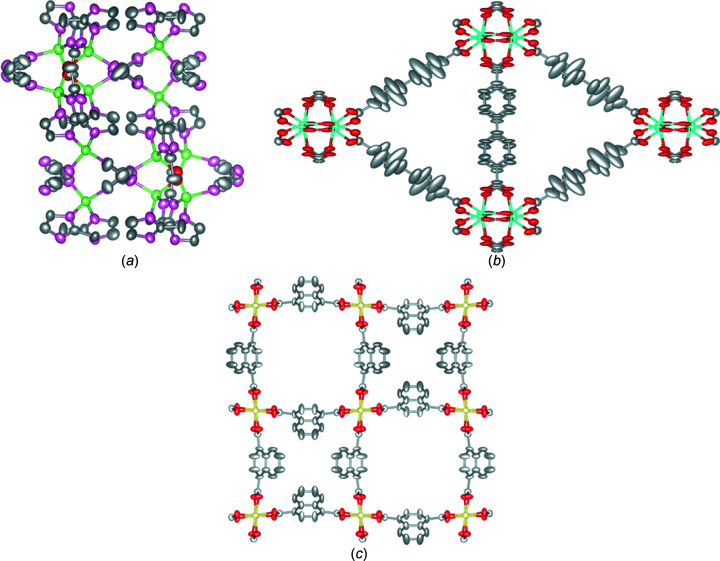
Ellipsoidal models of the investigated crystal structures (50% probability): (*a*) ZIF-EC1 along the *c* axis, (*b*) MIL-140C along the *c* axis and (*c*) Ga(OH)(1,4-ndc) along the *c* axis. Zn atoms are shown in green, Zr in cyan, Ga in yellow, O in red, N in pink and C in grey. H atoms have been omitted for clarity.

In this study, three MOFs were selected as examples: ZIF-EC1, MIL-140C and Ga(OH)(1,4-ndc) (1,4-ndcH_2_ is naph­tha­lene-1,4-dicarboxylic acid). A summary of the structural information and structure models is shown in Table 1 ▸ and Fig. 1 ▸. Although the structure models of these MOFs, obtained using cRED, have been published (Ge *et al.*, 2021*a* ▸; Samperisi *et al.*, 2021 ▸; Rabe *et al.*, 2020 ▸), the previous investigations focused mainly on the structure determination of these materials and the study of their properties. Except for MIL-140C, the study and interpretation of the ADPs in the structure models were not carried out. In addition, strategies for data processing against cRED data to obtain accurate ADPs have not been proposed. We selected these materials for their structural diversity, which allows more general conclusions to be drawn and the interconnection between the structural attributes of the frameworks and the obtained ADPs to be understood. Additionally, as these structures have been investigated previously in our laboratory, we have full access to the raw cRED data used for their original structure determination. In this section, we describe the structural details of the MOFs, specifying what has been published and which data sets from the previously published works are used in this study.

### 2.1. ZIF-EC1

The structure of ZIF-EC1 (Ge *et al.*, 2021*a* ▸) is constructed by Zn^2+^ cations and deprotonated 2-methyl­imidazole (mIm^−^) linkers [Fig. 1 ▸(*a*)]. The structure crystallizes in a monoclinic space group in which two Zn^2+^ cations are coordinated to three mIm^−^ linkers and one bridging OH^−^ group to form a binuclear Zn_2_N_6_(OH) cluster, while one Zn^2+^ cation coordinates four mIm^−^ linkers to form a ZnN_4_ mononuclear cluster. The structure is nonporous to N_2_.

ZIF-EC1 has been used previously to show that higher data completeness gives rise to models with more precise atomic positions (Ge *et al.*, 2021*b* ▸ ▸). An evaluation of the obtained models in terms of the ADPs has not been performed. The work used ten data sets collected from individual crystals of ZIF-EC1, with variable resolutions in the range 0.70–1.21 Å. Here we use the data sets with a resolution higher than 0.81 Å, which include six of the ten original data sets (Table 2 ▸).

### 2.2. MIL-140C

MIL-140C crystallizes with monoclinic crystal symmetry. It is built from Zr–oxo chains connected by bi­phenyl-4,4′-di­carb­oxy­late (bpdc) linkers to form one-dimensional (1D) microporous channels [Fig. 1 ▸(*b*)]. There are two crystallographically independent bpdc linkers in the structure. The linkers along the crystallographic *c* axis are stabilized through π–π interactions, while the linkers in the *ab* plane have more spatial freedom. The *ab initio* structure solution from 3D ED data, which revealed experimentally the linker motions in MIL-140C, has been reported by our group (Samperisi *et al.*, 2021 ▸). We showed that the linkers in the *ab* plane (linker 1) exhibit considerably more elongated displacement ellipsoids than the π-stacked linkers (linker 2). The elongation is caused by small amplitude librational motions around the linker axis, which agrees well with a study by ^2^H solid-state nuclear magnetic resonance (ssNMR) spectroscopy (Samperisi *et al.*, 2021 ▸).

In the previously published study, nine individual cRED data sets were merged into three groups to obtain three merged structures that were used for the analysis of the ADPs to probe the linker motions. A detailed evaluation of the different models obtained after refinement of the ADPs from the individual data sets was not reported. Additionally, in the previous study, restraints (SIMU and DELU in *SHELXL*; Sheldrick, 2015*b* ▸) were applied on the displacement parameters of the atoms in linker 2. In this study, we selected 19 data sets, including the nine investigated previously, with data resolutions of 0.79–0.87 Å (Table S4 in the supporting information).

### 2.3. Ga(OH)(1,4-ndc)

The crystal structure of Ga(OH)(1,4-ndc) crystallized in a tetragonal crystal system in which Ga(OH)_2_O_4_ octahedra are connected through naphthalene-1,4-di­carboxyl­ate (ndc) linkers. The framework has two square channels of different diameters [Fig. 1 ▸(*c*)].

In the previously published study (Rabe *et al.*, 2020 ▸), the crystal structure was solved from an individual crystal of the MOF. Although the refinement details obtained using cRED data were summarized in a table, the published structure was refined using the same isotropic displacement parameters for each element after Rietveld refinement against PXRD data. In this work, we use eight cRED data sets with a resolution of 0.80 Å, including the data set used previously to solve the structure (Table 5).

## 3. Data collection, processing and refinement

For all samples, the cRED experiments were performed on a JEOL JEM-2100-LaB6 at 200 kV (Cs 1.0 mm, point resolution 0.23 nm) equipped with a 512 × 512 Timepix hybrid pixel detector (55 µm × 55 µm pixel size, model QTPX-262k) from ASI. The software *Instamatic* (Cichocka *et al.*, 2018 ▸) was used for data collection. Detailed data collection information for each sample can be found in the literature (Ge *et al.*, 2021*b* ▸; Samperisi *et al.*, 2021 ▸; Rabe *et al.*, 2020 ▸).

Data reductions were performed using *XDS* (Kabsch *et al.*, 2010*a* ▸). For each structure, the individual data sets were indexed according to the space group and unit-cell parameters specified in Table 1 ▸. The cross-correlation coefficient between random half data sets (CC_1/2_) was used to estimate the resolution cut-offs of the diffraction data sets. The structures were solved *ab initio* with the dual-space algorithm implemented in *SHELXT* (Sheldrick, 2015*a* ▸), followed by a full-matrix least-squares refinement using a kinematical approximation by the *SHELXL* program (Sheldrick, 2015*b* ▸). The atomic coordinates of the individual data sets of ZIF-EC1 and Ga(OH)(1,4-ndc) were compared to the best individual data set available in our study; the individual data sets of MIL-140C were compared to the structure reported previously (Samperisi *et al.*, 2021 ▸).

To allow an easy comparison among the data sets, for each structure, one *SHELXL* input file was used for the refinement of all data sets. Details that are specific to the refinement of each structure are described in §5. Here we report the com­mon features. Atomic scattering factors based on neutral atoms and the wavelength for electrons were applied. All the non-H-atom positions were located directly from the initial structure solution. H atoms were generated geometrically by HFIX (Sheldrick, 2015*b* ▸). *XSCALE* (Kabsch *et al.*, 2010*b* ▸), part of the *XDS* package, was applied for data merging. The atomic displacement parameters were refined anisotropically from both individual and merged data sets. For the analysis and interpretation of the displacement parameters, no restraints or constraints were applied to the ADPs. The three eigenvalues λ1, λ2 and λ3 (Fig. S1 in the supporting information), calculated from the ADPs given in the *SHELXL* output file, were used for plotting the ADPs. The models obtained after refining the ADPs against the individual and merged data sets were compared. Additionally, for each MOF, different merging strategies were applied, and the obtained models with refined ADPs were compared to establish the best merging strategy. A detailed description of the refinement strategies applied is reported for each sample in §5. To evaluate the final models, we compared the difference in *R* values and ADPs. The criteria used to inspect the ADPs are discussed in §4.

## 4. Inspection of the displacement ellipsoids

The overall quality of a structure model is typically evaluated by agreement parameters (*R* values and goodness-of-fit *S*). The transition from isotropic to anisotropic doubles the number of parameters to refine and, therefore, restraints or constraints are commonly applied for refining ADPs. However, a visual inspection of the ellipsoids before imposing any restraints/constraints can provide information to evaluate the physical meaning of the ADPs and identify possible problems of various natures (*e.g.* incorrect structure, incorrect data treatment, *etc*.). *checkCIF*/*PLATON* (Spek, 2020 ▸) validation generates alerts when unusual ADPs are detected (*e.g.* when the ellipsoids are unreasonably flat). It is also of paramount importance to assess the correctness of a structure based on chemical and crystallographic knowledge. Therefore, to inspect the ellipsoidal models, in addition to the validation tests implemented in *checkCIF*/*PLATON*, we asked ourselves the following questions.

(i) Are the atoms NPD or the ellipsoids unreasonably flat? A displacement ellipsoid with one or more of the three half-axes refined to negative or almost zero values [*i.e.* nearly two-dimensional (2D)] are physically meaningless. A structure model containing atoms of this kind should not be deposited in a structure database. When NPD atoms are observed, it is recommended to find the reasons (*e.g.* wrong atom-type assignment, unresolved disorder, poor-quality crystals and/or data sets, *etc*.) and make further efforts to improve the data quality so that they allow a full anisotropic refinement (adding restraints/constraints, removing the atom and reintroducing it after some cycles of refinement).

(ii) Are the ellipsoids strongly elongated? Ideally, the displacement ellipsoids would be nearly spherical for atoms without disorder or displacement. Therefore, the *checkCIF*/*PLATON* validation tool checks whether the ratio of the maximum and minimum ADP components along three main axes deviates significantly from 1.0. Strong anisotropic behaviour (*i.e.* a large deviation from 1.0) should be investigated as it is likely that large disorder, inadequate treatment of the data or experimental errors are contributing to the displacement parameters. In general, if the displacement ellipsoids are elongated in one direction, it can indicate that these atoms have discrete conformations (displacive disorder) or move more strongly in that direction than in others (dynamic).

(iii) How similar are the ADPs of atoms belonging to the same molecular fragment? According to the Hirshfeld theorem (rigid-bond criterion) (Hirshfeld, 1977 ▸), the components of the ADPs of atoms along the bond direction should have similar values. It is also reasonable to expect that the ellipsoids of bonded atoms with the same chemical environment should be similar in both size and orientation. If these conditions are not fulfilled, the ellipsoidal model is inadequate for a physically sensible description of the crystal structure.

## 5. Results and discussion

For all samples, structure refinements were performed against individual and merged data sets. The deviations of atomic positions (Tables S1–S3 in the supporting information), complete refinement details (Tables 1–6 and Table S4 in the supporting information) and plots of the ADPs (Figs. S2–S4) are given either in the main text or as supporting information. Ellipsoidal models of the individual and merged data sets are used for visualizing and inspecting the trends of the ADPs, *i.e.* consistency in the anisotropy of the molecular fragments by the size, shape and orientation of the ellipsoids. For comparing the values of the ADPs among individual data sets, bar-chart representations of averaged eigenvalues (λ1, λ2 and λ3) and their standard deviations were calculated (Figs. S2–S4). Ellipsoidal models of the merged data sets are compared to gain insights into the effectiveness of the merging strategies. In this section, we describe and discuss the general observations and trends of each studied example and propose optimal strategies to model the structures.

### 5.1. ZIF-EC1

**Figure 2:**
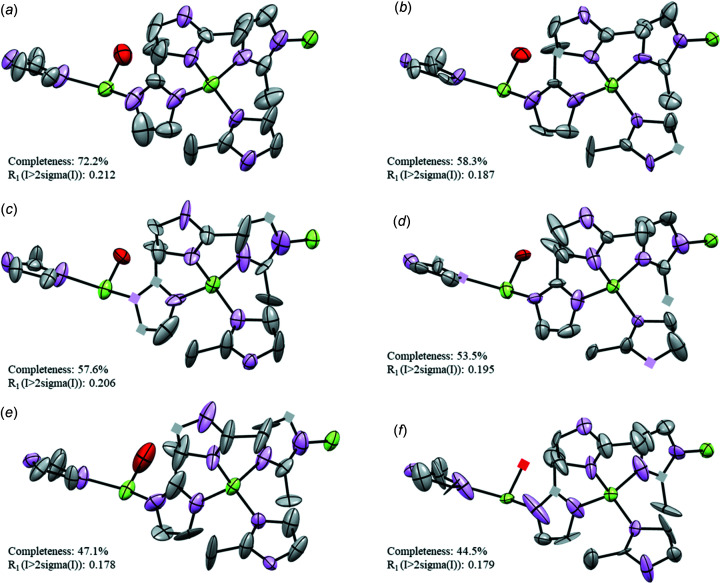
Ellipsoidal models of ZIF-EC1 obtained from the individual data sets. (*a*)–(*f*) Data sets 1–6, respectively. ADPs are shown at the 50% probability level. Cubes correspond to NPD atoms. Zn atoms are shown in green, O in red, N in pink and C in grey. H atoms have been omitted for clarity.

For the refinement of ZIF-EC1, soft geometrical restraints (DFIX and FLAT; Sheldrick, 2015*b* ▸) were applied to the mIm^−^ linkers to maintain a reasonable geometry. The deviations of the atomic positions given in Table S1 (see supporting information) show a good agreement of the atomic positions among the individual data sets [average deviation 0.06 (2) Å]. The individual data sets have a completeness that ranges from 44.5 to 72.2%, and the refinement results are comparable, as indicated by, for example, the similarity of the *R*_1_ values given in Table 2 ▸. The ellipsoidal models obtained from the individual data sets are shown in Fig. 2 ▸, where the models are presented in descending order of completeness (data sets 1–6).

**Figure 3:**
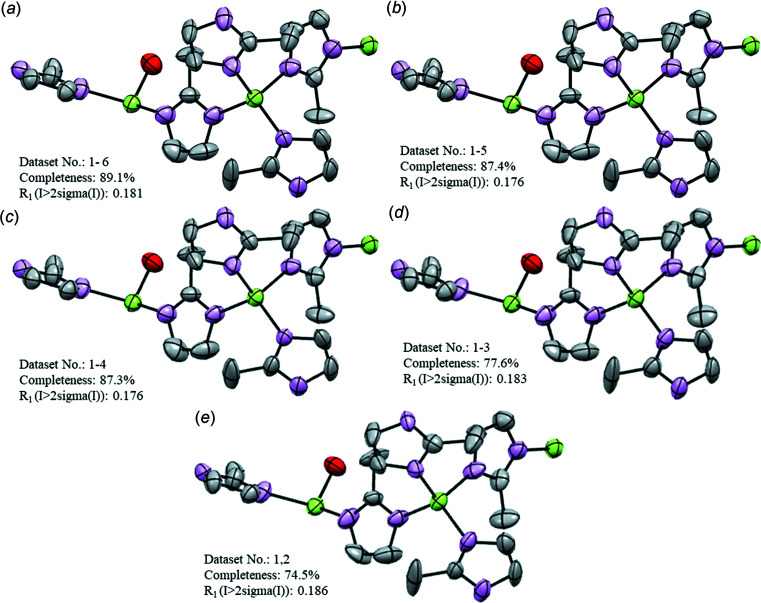
Ellipsoidal models of ZIF-EC1 obtained from the merged data sets. (*a*)–(*e*) Merged (m) data sets 1m to 5m, respectively. ADPs are shown at the 50% probability level. Zn atoms are shown in green, O in red, N in pink and C in grey. H atoms have been omitted for clarity.

For ZIF-EC1, despite some variations of the eigenvalues obtained from the different individual data sets, all six models show similar trends, where elongations of ellipsoids are observed for some atoms (Fig. 2 ▸ and Fig. S2 in the supporting information). However, the λ1 values are relatively small (λ1 ≤ 0.3 Å^2^) and do not suggest the presence of large atomic motions. The observation is in agreement with the nature of the framework structure. In this dense framework, the atoms have less spatial freedom than in a porous structure. This confers less mobility to the linkers. In data sets 2–6 [Figs. 2 ▸(*b*)–(*f*)], some ellipsoids are disk-like and the ADPs for some atoms are NPD due to negative values of the λ3 tensor. For all data sets, the ellipsoids of the atoms (*e.g.* the adjacent C atoms in the mIm^−^ ring) show a difference in orientation. This difference is obvious in data sets 2–6, but it is also observed in data set 1. Among all the data sets, the individual data set 1 with highest completeness (72.2%) undoubtedly provides the best structure model, as the ellipsoids are positively definite and more spherical (Fig. 2 ▸). Therefore, in an attempt to improve the final models, the data sets were merged to attain a higher completeness. A total of five attempts of data merging were performed. We started merging all the data sets together [data set 1m; Fig. 3 ▸(*a*)] and then we progressively removed the individual data sets with a lower individual completeness [data sets 2m–4m; Figs. 3 ▸(*b*)–(*d*)], until only the two individual data sets with the highest individual completeness remained and these were merged [data set 5m; Fig. 3 ▸(*e*)]. After merging, the completeness increased from 74.5 to 89.1%. After refinement, the merged data sets show similar statistics with comparable *R* values (Table 3 ▸). It is worth noting that the agreement parameters obtained after merging show a general improvement compared to those for the individual data sets. The ellipsoidal models improved significantly after merging. All the atoms refined to positive values of the tensors, no disk-like ellipsoids are detected and the ellipsoids of atoms belonging to the same molecular fragment are similar both in size and orientation. Additionally, the ellipsoids show a more isotropic behaviour, consistent with the nature of a framework where no significant motion occurs.

The models of ZIF-EC1 with refined ADPs benefited from the merging strategy used, as no appreciable difference in size, shape and orientation of the ellipsoids between the merged data sets are detected. The improvement observed for the model obtained from data set 5m compared to the model obtained from individual data set 1 is related to the en­hance­ment of data quality, *i.e.* lower *R*_1_ values, registered after merging. It should be noted that all the models shown in Fig. 3 ▸ are chemically sensible and can be deposited in a structure database, even though restraints/constraints were not applied to the ADPs.

### 5.2. MIL-140C

**Figure 4:**
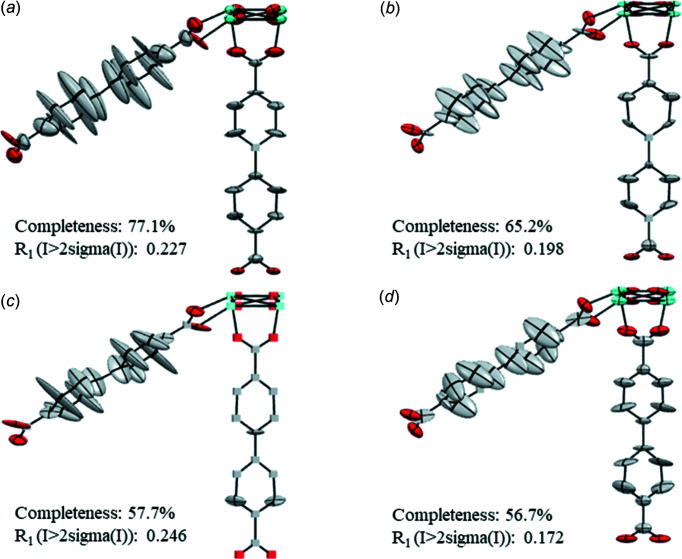
Ellipsoidal models of MIL-140C obtained from the individual data sets, *i.e.* (*a*) 1, (*b*) 11, (*c*) 2 and (*d*) 16. ADPs are shown at the 50% probability level. Cubes correspond to NPD atoms. Zr atoms are shown in cyan, O in red and C in grey. H atoms have been omitted for clarity.

**Figure 5:**
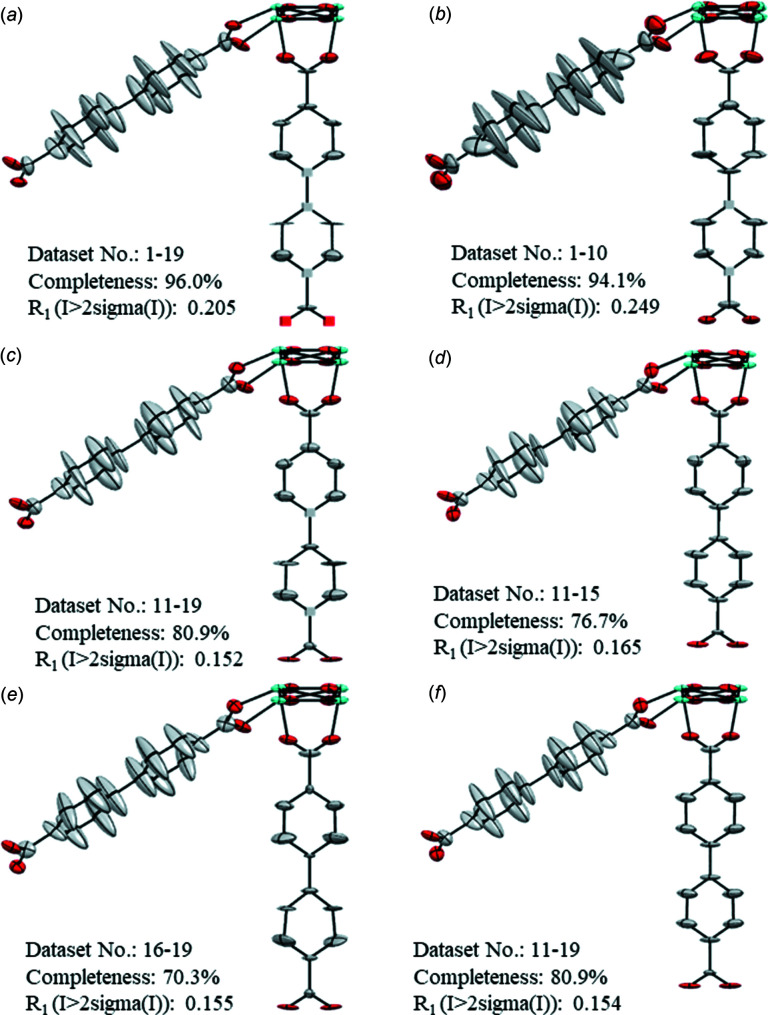
Ellipsoidal models of MIL-140C obtained from the merged data sets. (*a*)–(*e*) Merged (m) data sets 1m to 5m, respectively and (*f*) merged data set 3m after restraining and constraining the ADPs. ADPs are shown at the 50% probability level. Cubes correspond to NPD atoms. Zr atoms are shown in cyan, O in red and C in grey. H atoms have been omitted for clarity.

The refinement of MIL-140C was performed applying soft geometrical restraints (DFIX, DANG and FLAT; Sheldrick, 2015*b* ▸) to the dynamic fragment, linker 1 on the benzene rings. The SWAT (Langridge *et al.*, 1960 ▸) parameter was applied to all structures at the end of each refinement to compensate for the effects of solvent in the pores. The atomic coordinates of the 19 individual data sets were compared with the reference structure from the literature (Samperisi *et al.*, 2021 ▸). The atomic positions have an excellent agreement [average deviation 0.04 (2) Å], indicating the consistency among the 19 structure models (Table S2 in the supporting information). The completeness of the individual data sets ranges from 33.7 to 80.1%. As indicated by the variable *R* values, the individual data sets show different refinement statistics (Table S4), which is also reflected in the fluctuations of the eigenvalues (Fig. S3). Nevertheless, the ADPs of the 19 data sets show consistency in their general trend, as shown in Fig. 4 ▸, where the ellipsoidal models obtained from four randomly picked individual data sets are chosen to present this common trend. The models are presented in descending order of completeness. The ellipsoids of the C atoms of linker 1 are more elongated in the direction perpendicular to the ring plane than the ellipsoids of the π-stacked chain (linker 2). This trend reflects the dynamics of linker 1 reported previously (Samperisi *et al.*, 2021 ▸). In all data sets, λ3 for some atoms are refined to negative values or values close to zero. Interestingly, the atoms showing NPD behaviour are mostly in linker 2, which is more rigid than linker 1. Additionally, the ellipsoids of linker 1 tend to have a different size, shape and orientation. The frequently observed NPD behaviour of the atoms and the variable size, shape and orientation of the ellipsoids show that the refinement against individual data sets resulted in poor ellipsoidal models. Thus, it is hard to obtain detailed information about the structural details and the unique features of the structure. To improve the ellipsoidal models, we merged the data sets for refinement. The tables with the complete refinement details for the merged data sets are given in Table 4 ▸. At first, all the data sets were merged together as data set 1m [Fig. 5 ▸(*a*)]. Despite the higher completeness (96.0%) compared to the individual data sets, the obtained model does not show a significant improvement of the ellipsoids. Some atoms of linker 2 refine to negative values of λ3 or give rise to unreasonably flat ellipsoids. The ellipsoids of linker 1 are directional, consistently pointing from the linker ring planes, but flat along the bond directions. Considering the high number of individual data sets and their variable behaviours on refinement, a merging inclusive of all data sets might not be the best option to improve the ellipsoidal models. Thus, we evaluate a criterion which could be used to optimize the selection of the data sets. For the subsequent merging strategies, the individual data sets were grouped depending on the *R*_1_ values. The group 2m is composed by the data set with *R*_1_ > 0.20 from the individual data sets 1–10 [Fig. 5 ▸(*b*)]. The group 3m contains data sets 11–19, with *R*_1_ < 0.20 [Fig. 5 ▸(*c*)]. Both refinements generate models, with some atoms marked as NPD and flat ellipsoids in linker 2. However, despite the higher completeness compared to 3m, the refinement against 2m generates a model in which the ellipsoids of the atoms in linker 1 have different orientations. The result suggests that the exclusion of data sets with *R*_1_ > 0.20 leads to an improved model despite a lower completeness. Keeping this in mind, we further divided the individual data sets from 11 to 19 into two groups for merging. The first group, 4m, includes the individual data sets from 11–15, which have 0.18 < *R*_1_ < 0.20 [Fig. 5 ▸(*d*)]. The second group, 5m, is composed by the individual data sets with *R*_1_ < 0.18 from data sets 16–19 [Fig. 5 ▸(*e*)]. In both models, no atom shows NPD behaviour. However, the NPD atoms from the refinement against 3m become positively defined for 4m, yet was still unreasonably flat. The refinement against 5m gave rise to a model with slightly tilted ellipsoids in linker 1. Additionally, some ellipsoids in linker 2 appear too small compared to the neighbouring atoms.

Through a comparison of the ADPs among individual and merged data (Figs. 4 ▸ and 5 ▸), we can make several general conclusions. For MIL-140C, merging was beneficial for improving the final ellipsoidal model and is essential for obtaining crucial information about the motion of linker 1. After merging (Fig. 5 ▸), the ellipsoids of linker 1 are more consistent in their orientations and draw a clear picture of the out-of-plane motion of the linker. Additionally, less atoms are refined to negative values of the tensors or result in disk-like ellipsoids. Among all the merging strategies, the best results were obtained when grouping data sets with an *R*_1_ value below 0.20 [data set 3m; Fig. 5 ▸(*c*)], although the completeness is 80.9%. This result suggests that the quality of the individual data sets used for merging is reflected in the final models, especially on the ADPs. However, the refinement against 5m [completeness 70.3%; Fig. 5 ▸(*e*)] generates a model with slightly different sizes and orientations of the ellipsoids, caused by the significant drop in completeness.

Therefore, when a large number of data sets are available, the optimal merging strategy for improving the ellipsoidal models is to apply the criterion ‘quality over quantity’, *i.e.* selecting the individual data sets showing lower *R*_1_ values as long as a reasonable completeness [*e.g.* 80.9%, like in data set 3m; Fig. 5 ▸(*c*)] can be reached. However, the use of chemical knowledge and restraints/constraints could be required. An example is provided in Fig. 5 ▸(*f*), where we show that the structure obtained after refinement against 3m is significantly improved after restraining/constraining the ADPs using chemical knowledge (refinement details are included in Table 4 ▸). The equal ADP constraint (EADP) was applied to make the ADPs of the C atoms in linker 1 equal. The EADP constraint was applied to the atoms along the C8—C17 axis and to the atoms at the side of the axis, separately. The similar ADP restraint (SIMU) and the rigid-bond restraint (DELU) were applied to the off-axis C atoms in linker 2, to make the ADPs of the dynamic atoms more similar. Through these simple corrections it was possible to obtain a model with all atoms positively defined and the conditions mentioned in §4 fulfilled.

### 5.3. Ga(OH)(1,4-ndc)

The refinement of Ga(OH)(1,4-ndc) was performed applying the SWAT parameter at the end of refinement. Geometrical restraints were not used. The individual data sets provide good agreement of the atomic positions [with an average deviation of 0.03 (2) Å] (Table S3 in the supporting information) and similar refinement statistics (Table 5 ▸). There is no large variation of the values of λ1, λ2 and λ3 for the same atom (Fig. S4). Additionally, for all eight data sets, the structures obtained after refinement against cRED data show a comparable trend of the ellipsoids, specifically, elongations of the ellipsoids of atoms C6 and C3 (Fig. S5). The elongation is consistently directional along λ1, *i.e.* in the out-of-plane direction. Atoms C5, C4, C2 and C1 are progressively more tilted in the direction of the C1—C1 axis in a way that resembles the curvature of a motion. The square channels create enough free volume for the ndc linkers to rotate. Therefore, the observed elongation and tilting are compatible, with a motion around the axis connecting the carboxyl­ate groups. The motion of the ndc linker has been reported for isostructural Zn_2_(1,4-ndc)_2_, which was investigated by ssNMR spectroscopy (Horike *et al.*, 2009 ▸).

**Figure 6:**
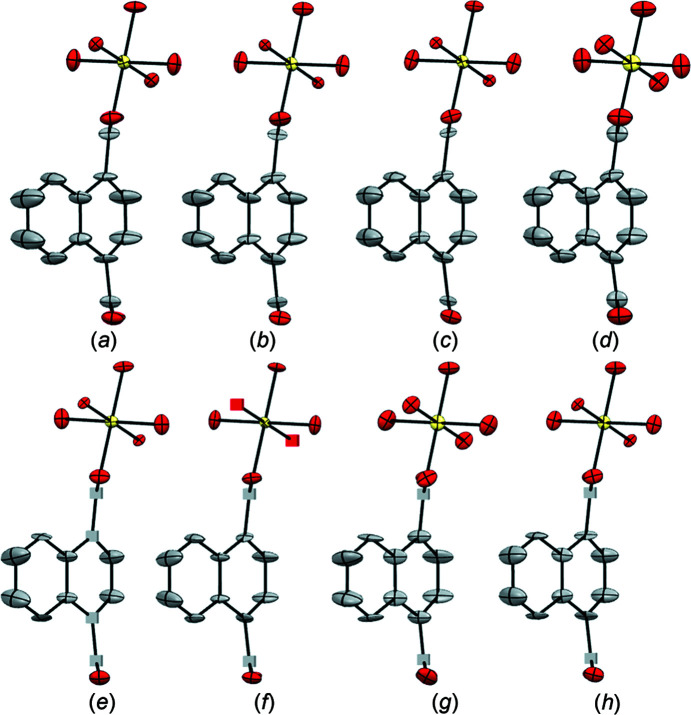
Ellipsoidal models of Ga(OH)(1,4-ndc) obtained from the individual data sets. (*a*)–(*h*) Data sets 1–8, respectively, with ADPs shown at the 50% probability level. Cubes correspond to NPD atoms. Ga atoms are shown in yellow, O in red and C in grey. H atoms have been omitted for clarity.

In the ellipsoidal models 1–4, all the atoms are positively defined and the ADPs are all reasonable [Figs. 6 ▸(*a*)–(*f*)]. Some atoms showing NPD behaviour can be observed in ellipsoidal models 5–8 [Figs. 6 ▸(*e*)–(*h*)]. The atoms marked as NPD are consistently observed for the C atoms along the axis of the arene ring connected to the metal nodes. For data set 6, the O atom in the Ga–oxo cluster is marked as NPD. All the obtained models show elongated ellipsoids of the less constricted C atoms able to undergo rotation. These ellipsoids are consistent in size, shape and orientation within each model. For all models, the trend of the ADPs is consistent and easy to interpret. Although in four models some atoms are refined to negative values of λ3, the linker flexibility can still be deduced from the elongation and tilting of the positive definite ellipsoids. However, models containing atoms marked as NPD, should not be deposited in a structure database and more investigation is needed. The model obtained from data set 1 was chosen for deposition in the Cambridge Structural Database (CSD; Groom *et al.*, 2016 ▸), as it has some of the lowest *R*_int_ and *R*_1_ values (Table 5 ▸). Considering the advantages of data merging for ZIF-EC1 and MIL-140C, we merged the data sets and investigated the final ADPs of Ga(OH)(1,4-ndc).

**Figure 7:**
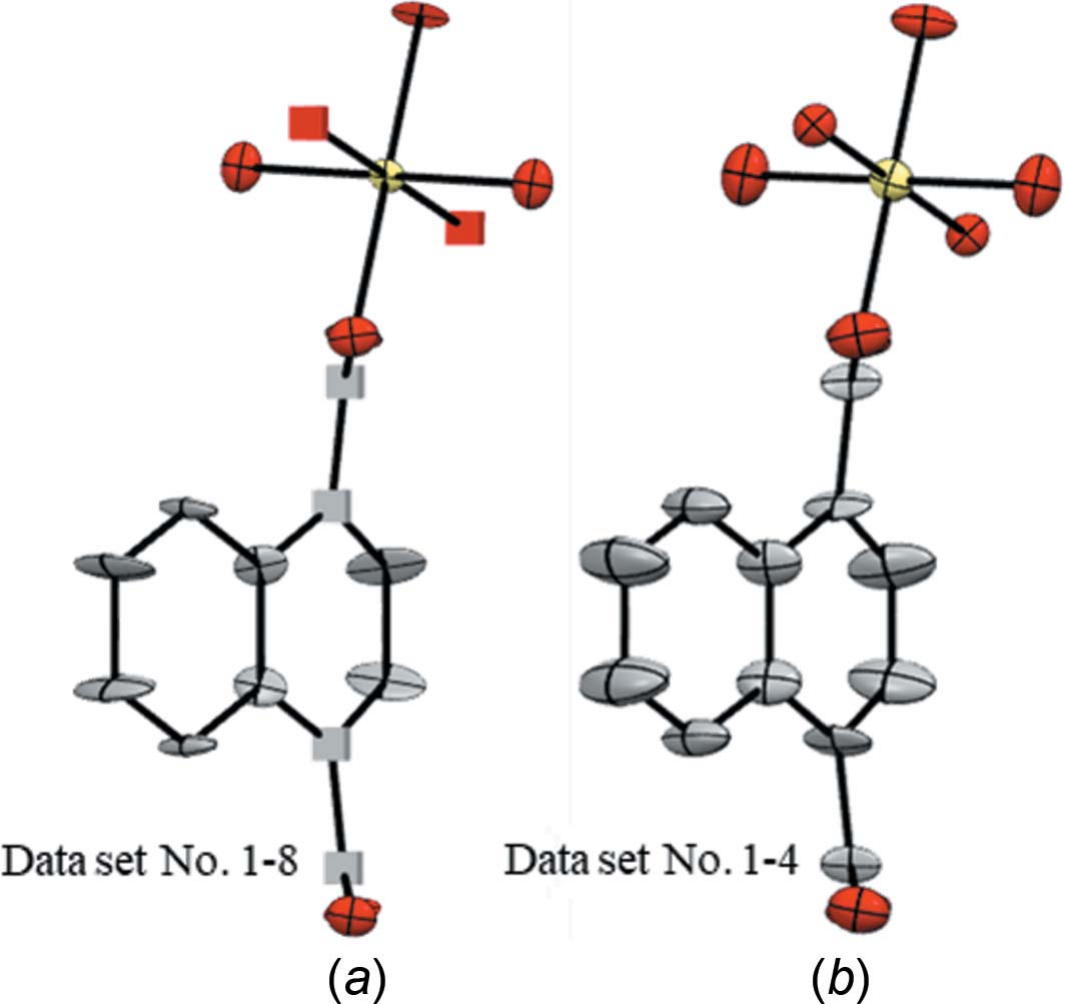
Ellipsoid models of Ga(OH)(1,4-ndc) obtained from the merged data sets, *i.e.* (*a*) 1m and (*b*) 2m. ADPs are shown at the 50% probability level. Cubes correspond to NPD atoms. Ga atoms are shown in yellow, O in red and C in grey. H atoms have been omitted for clarity.

Data sets 1–8 [1m; Fig. 7 ▸(*a*)] were merged in an attempt to improve the structure models. Data sets 1–4 [2m; Fig. 7 ▸(*b*)] were merged to test whether more structural details could be gained. Although the statistics show reasonable *R* values and completeness (Table 6 ▸), the ellipsoidal models obtained after refining against merged data show in one case a worse model (1m) and in the other case an almost equivalent model (2m) compared to the models obtained after refinement against individual data sets. Specifically, in model 1m, five atoms refined to negative values of λ3 and the remaining ellipsoids are unreasonably flat. Therefore, when individual data sets already provide the high completeness and high data-to-param­eter ratio required for a sustainable anisotropic refinement, merging does not prove to be a successful strategy to improve the ellipsoidal models. This is presumably because of the difference of each single crystal. Considering that the statistics from the individual data sets are almost equivalent, we assume that the refinement to negative tensors of some atoms in some data sets could be caused by an unreliable scaling factor, which is correlated to ADPs, rather than errors in the measured ED data or in the structure models. Our group is currently working on scaling strategies dedicated to ED data. At the moment, in such circumstances, to collect data from multiple crystals and refine them individually seems to be the best strategy for obtaining the best ellipsoidal models with no atoms refining to negative values of the tensors.

The structure of Ga(OH)(1,4-ndc) refined against data set 4 has been deposited in the CSD (CCDC reference: 2152260).

## 6. Conclusions

We analysed the structure models of three different MOFs and evaluated the ADPs obtained after refinement against 3D ED data. The data were collected by applying the cRED method and the refinement was performed by adopting kinematical approximation. We show that after the refinement of the ADPs, the resulting structure models can provide detailed information about linker motions, as can be identified in MIL-140C and Ga(OH)(1,4-ndc). Refinement against individual and merged data sets revealed that the ADPs in ZIF-EC1 and MIL-140C, which are low-symmetry crystals, benefited from merging multiple data sets. By evaluating the results for low-symmetry MOFs, it was possible to identify that both data completeness and data quality are reflected in the final ADPs and thus the structure models. The optimal strategy for obtaining most structural information from ADPs, therefore, consists in merging the individual data sets with the best refinement statistics, indicated by the *R*_1_ value, while maintaining a reasonable completeness. For Ga(OH)(1,4-ndc), which is a high-symmetry crystal, our results suggest that merging is not an optimal strategy to improve the ellipsoidal models and more work is currently in progress to overcome this limitation. As validation of ADPs obtained from 3D ED data is still in its infancy, further improvements are required when it comes to models used to describe the richness of ADPs in crystallographic refinement. We believe that the results presented herein will provide a starting point and motivate the community for further research. 

## Supplementary Material

Crystal structure: contains datablock(s) Ga-ndc_1. DOI: 10.1107/S2052252522005632/vq5001sup1.cif

Structure factors: contains datablock(s) Ga_1. DOI: 10.1107/S2052252522005632/vq5001Ga-ndc_1sup2.hkl

Zipped CIFs and structure factors. DOI: 10.1107/S2052252522005632/vq5001sup3.zip

Supporting Information. DOI: 10.1107/S2052252522005632/vq5001sup4.pdf

CCDC reference: 2152260

## Figures and Tables

**Table 1:** Crystal information for ZIF-EC1 (Ge *et al.*, 2021*a* ▸), MIL-140C (Samperisi *et al.*, 2021 ▸) and Ga(OH)(1,4-ndc) (Rabe *et al.*, 2020 ▸)

MOF	Crystal habit	Crystal system	Space group (No.)	No. unique atoms	*a*, *b*, *c* (Å)	α, β, γ (^o^)	Unit-cell volume, *V* (Å^3^)
ZIF-EC1	Plate-like	Monoclinic	*P*2_1_/*c* (14)	33	13.4619 (19), 14.659 (3), 14.449 (2)	90, 118.122 (11), 90.00	2514.7 (8)
MIL-140C	Plate-like	Monoclinic	*C*2/*c* (15)	23	32.360 (7), 15.8000 (3), 7.9100 (16)	90, 103.00 (3), 90	3940.6 (15)
Ga(OH)(1,4-ndc)	Rod shape	Tetragonal	*P*4_2_/*nmc* (137)	10	21.570 (3), 6.7000 (13)	90, 90, 90	3117.3 (11)

**Table 2:** Refinement details for the individual data sets of ZIF-EC1

Data set No.	1	2	3	4	5	6
Resolution (Å)	0.79	0.76	0.77	0.76	0.77	0.81
No. total reflections	9291	5092	5511	6502	5299	3935
No. unique reflections	3755	3427	3112	3132	2370	2189
No. unique reflections [*I* >2σ(*I*)]	2080	1897	1783	1874	1297	1495
Completeness (%)	72.2	58.3	57.6	53.5	47.1	44.5
*I*/σ	3.69	4.67	3.86	4.85	4.09	5.15
Redundancy	2.47	1.48	1.77	2.07	2.24	1.80
*R*_1_ [*I* > 2σ(*I*)]	0.212	0.187	0.206	0.195	0.178	0.179
*R*_1_ (all reflections)	0.250	0.239	0.247	0.236	0.233	0.210
Parameters	309	309	309	309	309	309
Restraints	45	45	45	45	45	45
*R*_int_	0.150	0.0691	0.113	0.0886	0.117	0.088
*wR*2	0.505	0.488	0.492	0.489	0.459	0.456
Goodness-of-fit	1.42	1.53	1.45	1.59	1.39	1.60

**Table 3:** Refinement details for the merged data sets of ZIF-EC1

Merged data set	1m	2m	3m	4m	5m
Individual data sets used for merging	All data sets	1–5	1–4	1–3	1–2
Resolution (Å)	0.77	0.77	0.77	0.77	0.77
No. total reflections	35748	31813	26518	20160	15007
No. unique reflections	5092	4998	4989	4434	4262
No. unique reflections [*I* > 2σ(*I*)]	3914	3739	3684	3127	2794
Completeness (%)	89.1	87.4	87.3	77.6	74.5
*I*/σ	5.54	5.18	4.83	4.74	4.47
Redundancy	7.02	6.37	5.31	4.55	3.52
*R*_1_ [*I* > 2σ(*I*)]	0.181	0.176	0.176	0.183	0.186
*R*_1_ (all reflections)	0.202	0.199	0.201	0.213	0.220
Parameters	309	309	309	309	309
Restraints	45	45	45	45	45
*R*_int_	0.168	0.169	0.166	0.166	0.148
*wR*2	0.472	0.438	0.438	0.452	0.456
Goodness-of-fit	1.68	1.19	1.17	1.21	1.21

**Table 4:** Refinement details for the merged data sets of MIL-140C

Merged data set	1m	2m	3m	4m	5m	3m_restrained
Individual data sets used for merging	All (1–19)	1–10	11–19	11–15	16–19	11–19
Resolution (Å)	0.80	0.80	0.80	0.80	0.80	0.80
No. total reflections	80052	44278	35667	22018	13544	35667
No. unique reflections	3874	3797	3263	2933	2835	3263
No. unique reflections [*I* > 2σ(*I*)]	3042	2867	2386	2067	1987	2386
Completeness (%)	96.0	94.1	80.9	76.7	70.3	80.9
*I*/σ	6.83	5.46	6.43	5.31	5.49	6.43
Redundancy	20.7	11.7	10.9	7.51	4.78	10.9
*R*_1_ [*I* > 2σ(*I*)]	0.205	0.249	0.152	0.165	0.155	0.154
*R*_1_ (all reflections)	0.228	0.269	0.188	0.207	0.191	0.190
Parameters	186	186	186	186	186	156
Restraints	17	17	17	17	17	31
*R*_int_	0.318	0.310	0.211	0.212	0.156	0.211
*wR*2	0.514	0.580	0.441	0.452	0.438	0.448
Goodness-of-fit	1.97	2.14	1.62	1.58	1.55	1.64

**Table 5:** Refinement details for the individual data sets of Ga(OH)(1,4-ndc)

Data set No.	1	2	3	4	5	6	7	8
Resolution (Å)	0.80	0.80	0.80	0.80	0.80	0.80	0.80	0.80
No. total reflections	12124	12518	13118	12619	12064	13155	12713	12189
No. unique reflections	1671	1683	1687	1664	1664	1664	1684	1679
No. unique reflections [*I* > 2σ(*I*)]	1288	1233	1166	1031	1290	1313	1160	1150
Completeness (%)	99.0	99.7	99.9	98.6	98.6	98.6	99.8	99.5
*I*/σ	6.13	4.71	4.21	5.02	5.15	5.28	6.35	4.92
Redundancy	7.26	7.45	7.75	7.57	7.26	7.91	7.49	7.30
*R*_1_ [*I* > 2σ(*I*)]	0.188	0.199	0.213	0.188	0.210	0.190	0.221	0.184
*R*_1_ (all reflections)	0.207	0.228	0.248	0.247	0.233	0.212	0.252	0.214
Parameters	86	86	86	86	86	86	86	86
Restraints	0	0	0	0	0	0	0	0
*R*_int_	0.164	0.229	0.237	0.213	0.218	0.212	0.205	0.208
*wR*2	0.471	0.483	0.480	0.489	0.495	0.452	0.532	0.447
Goodness-of-fit	1.71	1.61	1.55	1.57	1.71	1.57	1.85	1.47

**Table 6:** Refinement details for the merged data sets of Ga(OH)(1,4-ndc)

Merged data set	1m	2m
Individual data sets used for merging	All data sets	1–4
Resolution (Å)	0.80	0.80
No. total reflections	100473	50368
No. unique reflections	1688	1688
No. unique reflections [*I* > 2σ(*I*)]	1672	1635
Completeness (%)	100	100
*I*/σ	11.88	9.47
Redundancy	43.0	7.30
*R*_1_ [*I* > 2σ(*I*)]	0.205	0.206
*R*_1_ (all reflections)	0.211	0.208
Parameters	86	86
Restraints	0	0
*R*_int_	0.249	0.235
*wR*2	0.558	0.548
Goodness-of-fit	2.64	2.47
