# Supplementary material for: How to get maximum structure information from anisotropic displacement parameters obtained by three-dimensional electron diffraction: an experimental study on metal–organic frameworks
Source: IUCrJ. 2022 Jun 8;9(Pt 4):480–91. doi: 10.1107/S2052252522005632 (PMC9252158; doi:10.1107/S2052252522005632)
Supplement: Supplementary file 4 [file m-09-00480-sup4.pdf]

IUCrJ

Volume 9 (2022)

Supporting information for article:

How to get maximum structure information from anisotropic displacement parameters obtained by three-dimensional electron diffraction: an experimental study on metal–organic frameworks

Laura Samperisi, Xiaodong Zou and Zhehao Huang

How to Get Maximum Structure Information from Anisotropic Displacement Parameters Obtained by Three-Dimensional Electron Diffraction: An Experimental Study on Metal-Organic Frameworks

Laura Samperisi[a], Xiaodong Zou[a] and Zhehao Huang[a]

[a]Department of Materials and Environmental Chemistry, Stockholm University, Stockholm, Sweden, 106 91, Sweden

Correspondence email: xzou@mmk.su.se; zhehao.huang@mmk.su.se

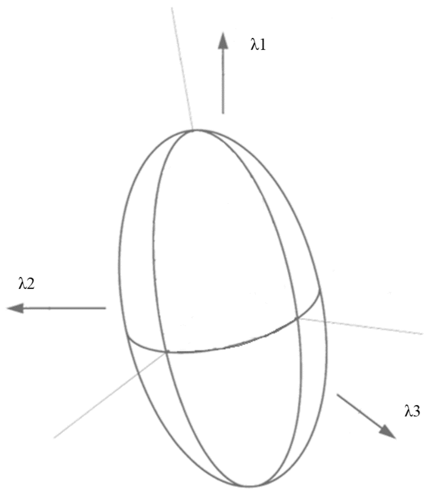

Figure S1 The ellipsoid model used to represent anisotropic atomic displacement, with major axes indicated. The ellipsoid is drawn with a specified probability of finding an atom inside its contour. The three eigenvalues calculated from the ADPs describe the ellipsoid: λ1 perpendicular to the bond, λ3 along the bond and λ2 at an arbitrary direction at right angle to λ3.

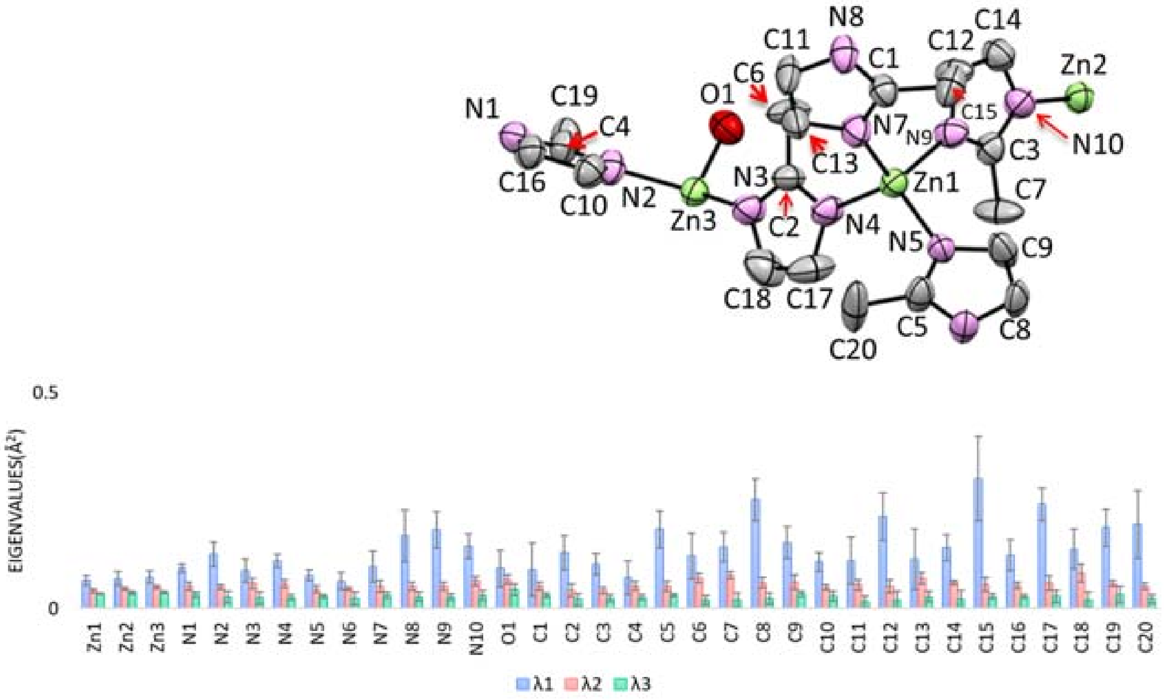

Figure S2 Average eigenvalues and their standard deviations of the individual datasets of ZIF-EC1. The inset is the ellipsoidal model of ZIF-EC1 obtained from dataset 1m, with atom labels. ADPs are shown at the 50% probability level. The values were averaged arithmetically and the standard deviations were calculated as the square root of the variance, including negative values whenever an atom was refined to negative values of the tensors. Zn are shown in green, O in red, N in pink and C in grey. Hydrogen atoms are removed for clarity.

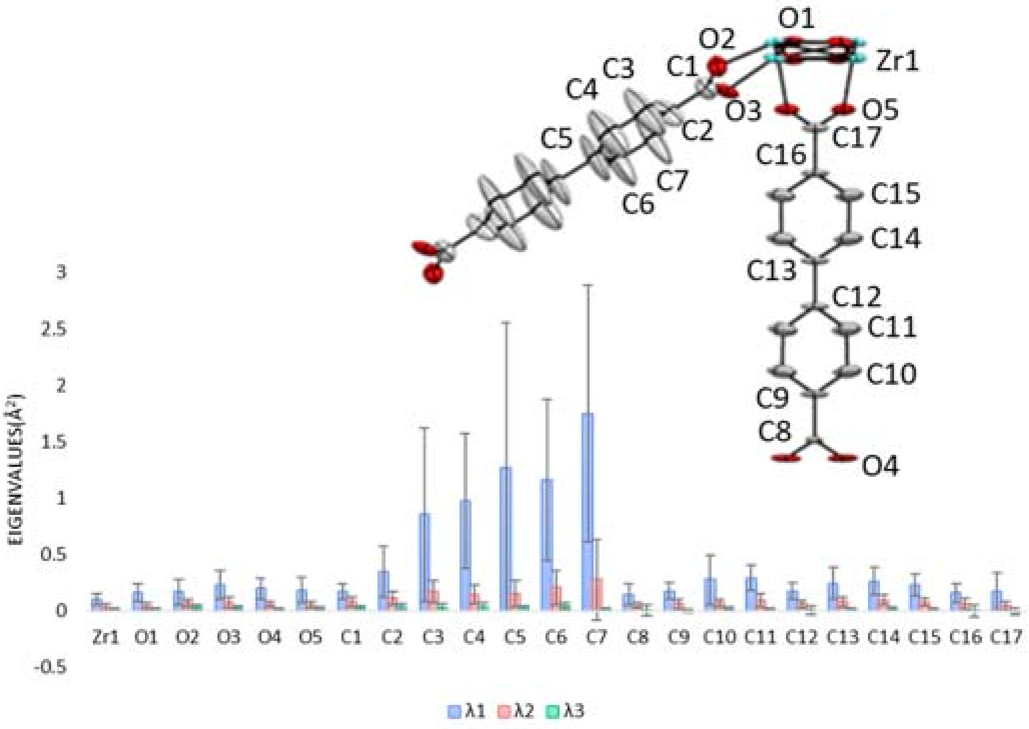

Figure S3 Average eigenvalues and their standard deviations of the individual datasets of MIL-140C. The values were averaged arithmetically and the standard deviations were calculated as the square root of the variance, including negative values whenever an atom was refined to negative values of the tensors. The inset is the ellipsoidal model of MIL-140C obtained from dataset 3m_restrained, with atom labels. ADPs are shown at the 50% probability level. Zr are shown in cyan, O in red and C in grey. Hydrogen atoms are removed for clarity.

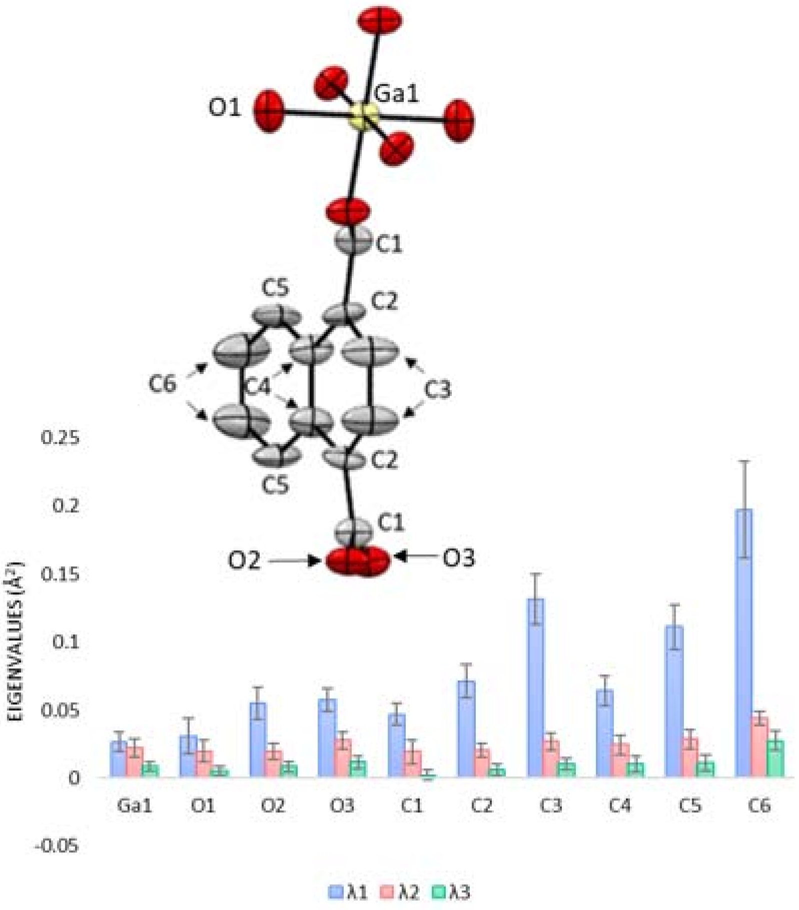

Figure S4 Average eigenvalues and their standard deviations of the individual datasets of Ga(OH)(1,4-ndc) . The values were averaged arithmetically and the standard deviations were calculated as the square root of the variance, including negative values whenever an atom was refined to negative values of the tensors. The inset is the ellipsoidal model of Ga(OH)(1,4-ndc) obtained from dataset 4, with atom labels. ADPs are shown at 50% probability level. Ga in yellow, O in red and C in grey. Hydrogen atoms are removed for clarity.

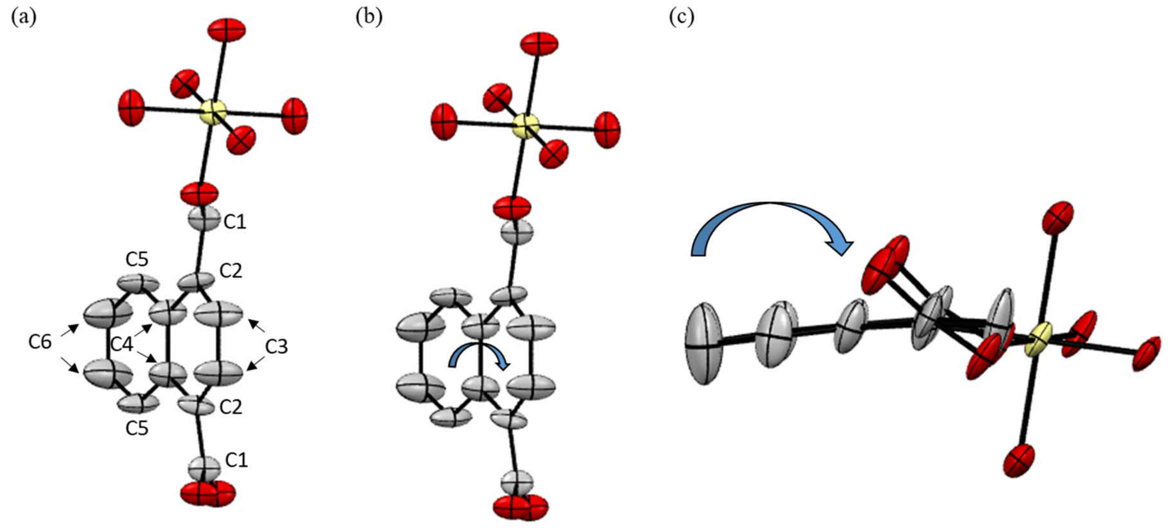

Figure S5 (a) Ga(OH)(1,4-ndc) along the c-axis with labelled atoms; (b) and (c) illustrate the linker dynamic in Ga(OH)(1,4-ndc). The ellipsoids have an inclination consistent with the linker motion. While C6, C5 and C3 point outside the linker plane, C1, C2 and C4 are slightly tilted according to the curvature of the motion in the direction of the carboxylate groups. ADPs are shown at 50% probability level. Ga in yellow, O in red and C in grey. Hydrogen atoms are removed for clarity.

Table S1 Average deviations of the atomic positions among the structure models obtained from individual datasets of ZIF-EC1. The deviations were calculated by comparing to a reference structure model obtained from the individual dataset 1.

Atom	Atomic coordinates deviation (Å)
Zn1	0.020(11)
Zn2	0.018(17)
Zn3	0.019(8)
N1	0.055(20)
N2	0.05(3)
N3	0.070(19)
N4	0.04(3)
N5	0.037(6)
N6	0.047(14)
N7	0.06(3)
N8	0.06(4)
N9	0.048(17)
N10	0.07(4)
O1	0.05(2)
C1	0.06(5)
C2	0.06(2)
C3	0.073(20)
C4	0.0254(10)
C5	0.038(18)
C6	0.09(4)
C7	0.07(2)
C8	0.049(10)
C9	0.034(11)
C10	0.07(3)
C11	0.06(5)
C12	0.040(8)
C13	0.06(4)
C14	0.06(5)
C15	0.13(11)
C16	0.05(2)
C17	0.06(3)
C18	0.07(2)
C19	0.07(3)
C20	0.06(3)
Average	0.06(2)

Table S2 Average deviations of the atomic positions among the structure models obtained from the individual datasets of MIL-140C. The deviations were calculated by comparing to the reference structure deposited from a previous study (Samperisi *et al.,* 2010, CCDC entry: 2073255).

Atom	Atomic coordinates deviation (Å)
Zr1	0.011(7)
O1	0.04(3)
O2	0.04(2)
O3	0.04(4)
O4	0.03(2)
O5	0.031(17)
C1	0.04(3)
C2	0.07(3)
C3	0.06(3)
C4	0.08(5)
C5	0.06(6)
C6	0.07(4)
C7	0.06(4)
C8	0.016(15)
C9	0.015(10)
C10	0.05(3)
C11	0.07(4)
C12	0.014(13)
C13	0.025(19)
C14	0.05(4)
C15	0.048(20)
C16	0.015(10)
C17	0.014(13)
Average	0.04(2)

Table S3 Average atomic coordinates deviation among the individual datasets of Ga(OH)(1,4-ndc). The deviations were calculated by comparing to a reference structure model obtained from the individual dataset 4. Ga1 and O1 occupy a special position.

Atom	Atomic coordinates deviation (Å)
Ga1	0
O1	0
O2	0.021(5)
O3	0.035(8)
C1	0.012(8)
C2	0.029(10)
C3	0.025(8)
C4	0.023(9)
C5	0.030(12)
C6	0.07(2)
Average	0.03(2)

Table S4 Refinement statistics for individual datasets of MIL-140C

Dataset no.	1	2	3	4	5	6	7	8	9	10	11	12	13	14	15	16	17	18	19
Resolution (Å)	0.79	0.84	0.84	0.83	0.81	0.87	0.80	0.80	0.80	0.80	0.83	0.83	0.85	0.81	0.83	0.83	0.87	0.81	0.79
No. of Total reflections	5458	3325	4196	4171	3558	4257	4945	3574	5468	5602	4939	2813	4546	5068	5591	4783	2510	2484	4010
No. Unique reflections	3157	1956	1469	2016	1464	1962	2021	2201	3186	2360	2256	1593	2094	2158	2421	2010	1248	1333	1982
No. Unique reflections (I >2sigma(I))	2024	926	1031	1137	1008	1444	1084	1179	1495	958	1510	1116	1243	1334	1429	1203	921	1118	1263
Completness	77.1%	57.7%	41.7%	55.1%	40.1%	63.2%	49.6%	54.1%	80.1%	58.7%	65.2%	45.8%	63.5%	57.7%	64.5%	56.7%	40.4%	33.7%	49.1%
I/σ	4.79	2.73	5.50	3.49	3.71	7.3	2.94	3.4	3.58	2.91	3.94	4.64	3.96	3.5	5.21	5.19	6.22	7.11	4.66
R meas	0.112	0.222	0.125	0.163	0.207	0.840	0.232	0.154	0.113	0.152	0.179	0.127	0.154	0.195	0.113	0.109	0.920	0.920	0.1240
Redundancy	1.73	1.70	2.86	2.07	2.43	2.17	2.45	1.62	1.72	2.37	2.19	1.77	2.17	2.35	2.13	2.38	2.01	1.86	2.02
R_1 (I>2sigma(I))	0.227	0.246	0.268	0.285	0.270	0.288	0.259	0.260	0.260	0.214	0.198	0.190	0.1853	0.189	0.195	0.172	0.168	0.168	0.169
R_1 (all reflections)	0.259	0.305	0.288	0.321	0.303	0.304	0.304	0.309	0.317	0.276	0.227	0.228	0.227	0.226	0.243	0.217	0.189	0.180	0.201
Parametere	186	186	186	186	186	186	186	186	186	186	186	186	186	186	186	186	186	186	186
Restraints	16	16	16	16	16	16	16	16	16	16	16	16	16	16	16	16	16	16	16
Rint	0.0802	0.162	0.103	0.129	0.166	0.0644	0.185	0.110	0.0805	0.116	0.141	0.0970	0.121	0.154	0.110	0.0873	0.0712	0.0713	0.0936
wR2	0.560	0.562	0.607	0.590	0.581	0.635	0.581	0.605	0.590	0.517	0.483	0.523	0.486	0.473	0.521	0.477	0.429	0.452	0.432
GooF	1.919	1.414	2.381	1.819	1.864	2.721	1.644	1.808	1.756	1.364	1.513	1.900	1.517	1.392	1.741	1.652	1.642	1.869	1.419

References

Samperisi, L.; Jaworski, A.; Kaur, G.; Lillerud, K. P.; Zou, X. & Huang Z. (2021). J. Am. Chem. Soc. **143**, 43, 17947–17952.
